# Which Is a More Accurate Predictor in Colorectal Survival Analysis? Nine Data Mining Algorithms *vs.* the TNM Staging System

**DOI:** 10.1371/journal.pone.0042015

**Published:** 2012-07-25

**Authors:** Peng Gao, Xin Zhou, Zhen-ning Wang, Yong-xi Song, Lin-lin Tong, Ying-ying Xu, Zhen-yu Yue, Hui-mian Xu

**Affiliations:** 1 Department of Surgical Oncology and General Surgery, The First Hospital of China Medical University, Shenyang, P.R. China; 2 Department of Gynecology and Obstetrics, Shengjing Hospital of China Medical University, Shenyang, P.R. China; The Chinese University of Hong Kong, Hong Kong

## Abstract

**Objective:**

Over the past decades, many studies have used data mining technology to predict the 5-year survival rate of colorectal cancer, but there have been few reports that compared multiple data mining algorithms to the TNM classification of malignant tumors (TNM) staging system using a dataset in which the training and testing data were from different sources. Here we compared nine data mining algorithms to the TNM staging system for colorectal survival analysis.

**Methods:**

Two different datasets were used: 1) the National Cancer Institute's Surveillance, Epidemiology, and End Results dataset; and 2) the dataset from a single Chinese institution. An optimization and prediction system based on nine data mining algorithms as well as two variable selection methods was implemented. The TNM staging system was based on the 7^th^ edition of the American Joint Committee on Cancer TNM staging system.

**Results:**

When the training and testing data were from the same sources, all algorithms had slight advantages over the TNM staging system in predictive accuracy. When the data were from different sources, only four algorithms (logistic regression, general regression neural network, Bayesian networks, and Naïve Bayes) had slight advantages over the TNM staging system. Also, there was no significant differences among all the algorithms (p>0.05).

**Conclusions:**

The TNM staging system is simple and practical at present, and data mining methods are not accurate enough to replace the TNM staging system for colorectal cancer survival prediction. Furthermore, there were no significant differences in the predictive accuracy of all the algorithms when the data were from different sources. Building a larger dataset that includes more variables may be important for furthering predictive accuracy.

## Introduction

Colorectal cancer is the third most common form of cancer for both males and females in the Western world and it has been estimated to account for more than 49,000 deaths in the United States in 2008 [1]. Predicting the outcome of cancer on the basis of clinical information is an important and challenging task in clinical practice. To our knowledge, the TNM classification of malignant tumors (TNM) staging system provided by the American Joint Committee on Cancer (AJCC), which is regarded as the strongest prognostic system for patients with colorectal cancer [2], is the technique that has been most widely used for this purpose. However, for colorectal cancer, the TNM staging system only involves three variables (primary tumor, regional lymph nodes, and distant metastasis) regardless of the N1c category, which is classified according to the tumor deposit, although it has been recommended that the TNM staging system should collect more prognostic factors [3], [4]. People will find themselves overwhelmed with dozens of parameters when more prognostic variables are included because standard statistics do not generally work in this situation [5]. For this reason, data mining, which is suitable for colorectal cancer survival prediction using past complex datasets, was applied to this field.

As early as 1997, Burke et al. indicated that artificial neural networks (ANNs), a back propagation network (BP) or multilayer perceptrons (MLPs) are significantly more accurate than the TNM staging system for colorectal cancer [6]. Subsequently, some authors also described the value of ANNs, classification and regression tree (CART) analysis, as well as logistic regression (LR) in predicting outcomes of colorectal cancer [7]–[11]. However, nearly all the studies used the same database to establish the prediction model and evaluate its value. To our knowledge, only Bottaci et al. used different datasets in the “learn” and “test” sections. Both the dataset for “learn” and prediction, however, were quite small with 334 items for “learn” and 92 for testing. There were several defects as well as, such as not being compared with TNM staging and there was only one algorithm was implemented [7]. Therefore, it still needs to be elucidated whether these models are really accurate in clinical practice when the patient data come from a different database.

Furthermore, there are many algorithms in the data mining family, such as support vector machines (SVM), adaptive-network-based fuzzy inference system (ANFIS), and Bayesian networks (BNs). Some of these algorithms have been used for survival prediction in other cancers besides colorectal cancer and comparisons among several of them have been made [11]–[13]. Nevertheless, nearly all such comparisons included no more than three algorithms and whether these methods are suitable for colorectal cancer is unknown.

The aim of our study was to analyze whether colorectal cancer survival prediction models built by nine algorithms together with two variable selection methods from a public database can be used with a relatively large private database with 760 >5-year follow-up cases that reflected the clinical practical value more accurately. We propose a synthetic scheme based on several techniques of data mining for predicting the outcome of colorectal cancer and compare them with the 7^th^ TNM staging system.

## Materials and Methods

### Data

The public dataset we used was the National Cancer Institute's Surveillance, Epidemiology, and End Results (SEER) dataset, 1973–2007. We chose patients diagnosed between 1998 and 2000. There were more than 200 variables in the SEER dataset. We selected twenty variables for analysis and the details are shown in Table 1. We did not change any data except that the “*AJCC stage 3^rd^*” was recoded as “*AJCC stage 7^th^*” according to the 7^th^ AJCC staging rules [3], [4]. From the selected cases, patients were extracted using the following criteria: 1) died of colon carcinoma in the 5 years after treatment; 2) alive after 5 years or more from time of diagnosis; and 3) without missing values. After using these data cleansing and data preparation strategies, the dataset, which consisted of 36,388 records was constructed. Additionally, we randomly selected 10,000 cases to form the final dataset. Of the 10,000 cases, 2000 were randomly selected for testing and the remaining 8000 were used for training.

**Table 1:** Variables Available for Analysis.

Variable	Type	Explanation	Supported
Inputs			
Age at diagnosis	Numeric	Years	Both^d^
Race/ethnicity	Categorical	22 races	Both
Sex	Binary	Female/male	Both
Primary Site	Categorical	Eleven sites	Both
AJCC^a^ stage 7^th^	Categorical	Pathologic code of TNM	Both
Grade	Categorical	Tumor grade (Grades 1–4)	Both
EOD^b^ 10 - size	Numeric	Size of primary tumor	Both
EOD 10 - extent	Categorical	Invasive extension of primary tumor	Both
EOD 10 - nodes	Categorical	Extension of lymph node involvement	SEER^e^
Regional nodes examined	Numeric	No. of regional lymph nodes examined	Both
Regional nodes positive	Numeric	No. of positive regional lymph nodes	Both
SEER historic stage A	Categorical	A stage system coded by SEER [15]	SEER
SEER summary stage 1977	Categorical	A stage system coded by SEER [15]	SEER
Histologic Type ICD-O-3^c^	Categorical	International Classification of Diseases for Oncology Third Revision	SEER
Number of primaries	Numeric	Number of primaries	Both
First malignant primary indicator	Binary	Yes/no	SEER
Radiation sequence with surgery	Categorical	Prior to/after surgery/both	SEER
Surgery of primary site	Categorical	Extension of surgery	Both
Surgery of oth reg/dis sites	Categorical	Surgery of other regional site(s), distant site(s), or distant lymph node(s)	Both
Outcome			
SEER cause-specific death classification	Binary	Yes/no	Both

AJCC^a^: American Joint Committee on Cancer.

EOD^b^: SEER extent of disease.

ICD-O-3^c^: International Classification of Diseases for Oncology Third Revision.

Both^d^: Supported by both SEER dataset and CMU-SO dataset.

SEER^e^: National Cancer Institute's Surveillance, Epidemiology, and End Results.

The private dataset (CMU-SO dataset) for testing included clinical information on all patients with colorectal cancer that underwent surgery at the Department of Surgical Oncology at the First Hospital of China Medical University from April 1994 to December 2007. Follow-up was completed for the entire study population until November 2008. More details about this dataset can be seen in our prior study [14]. Of 1541 patients, 760 were extracted according to the criteria used in the preparation stages of the SEER dataset and the remaining cases had >5-years follow-up. Another important work was mapping the primary data into the SEER dataset format. This process was strictly controlled according to the coding standard provided by SEER [15]. There were 14 variables of clinical pathological factors in the CMU-SO dataset and six variables selected from the SEER dataset were not supported by the CMU-SO dataset: *EOD 10 - nodes*, *SEER historic stage A*, *SEER summary stage 1977*, *Histologic Type ICD-O-3*, *Number of primaries*, *First malignant primary indicator*, and *Radiation sequence with surgery* (Table 1).

### Ethics statement

The study was approved by the Research Ethics Committee of China Medical University, China. Written informed consent was obtained from all patients before participating in the study. We have got permission to access the research data file in SEER program.

### Prediction Models

The TNM staging system used in this analysis was the pathologic system based on the 7^th^ edition of the AJCC TNM staging system and we considered stage IV as a whole entity, the same as the survival analysis made by AJCC [3].

Data mining is the process of extracting patterns from large datasets by using statistical methods or machine learning algorithms. It allows computers to “learn” from past examples and to detect hard-to-discern patterns from large, noisy or complex datasets. This capability is particularly suitable for predicting colorectal cancer survival [5]. To conduct an all-sided and scientific evaluation of a data mining technique being used for survival prediction, we used nearly all of the common algorithms. The algorithms used in this work included the BP network, radial basis function (RBF) neural network, general regression neural network (GRNN), ANFIS, SVMs, Naïve Bayes (NB), BNs, CART, and LR. To ensure the reproducibility of our work, some details about these algorithms are presented in Table 2. Furthermore, to increase the predictive accuracy, several parameters were determined by an optimization and prediction system.

**Table 2:** Nine algorithms used in the construction of prediction models.

Data mining algorithm	Features	Details	Optimized parameters	Reference
BP	one kind of artificial neural networks which is used most frequently	A single hidden layer was used and Levenberg-Marquardt backpropagation was used as a backpropagation network1000 cases derived from the training cases were chosen for validation to avoid “overfitting”	the number of neurons in the hidden layer	Burke [6]Bottaci [7]Snow [8]Grumett [9]Delen [16]
CART	easy to understand and efficient training algorithm	1000 cases divided from training cases were chosen for pruning		Valera [10]Schwarzer [13]
SVM	more robust and overfitting is unlikely to occur	The kernel function used in this study was RBF	cost (c) which controls overfitting of the model, and gamma (g), which controls the degree of nonlinearity of the model	Tanabe [17]
ANFIS	a fuzzy inference system in the framework of a multilayer feed-forward network	subtractive clustering and the hybrid learning algorithm were used to generate the fuzzy inference system and the parameter estimation of membership function		Schwarzer [13]Catto [18]
RBF	an alternative to the BP network which has a radial basis layer			Venkatesan [19]Bardan [20]
GRNN	similar to RBF and has a special linear layer after the radial basis layer		the width of the RBF, denoted as the spread	Lai [21]Naguib [22]
LR	has a good accuracy and fast development timebased on the linear assumption			Schwarzer [13]Bartfay [23]
NB	easy to understand and efficienttraining algorithm which assumes attributes are statistically independent			Friedman [24]Witten [25]
BNs	a algorithm based on Bayes' theorem and represents conditional dependencies of variables via a directed acyclic graph	maximum likelihood parameter estimation was used for the parameter learning	the structure learning method was chosen among three algorithms including K2, Markov Chain Monte Carlo and tree augmented Naïve Bayesian	Jayasurya [12]Friedman [24]Witten [25]

### Optimization and Prediction System

**Figure 1:**
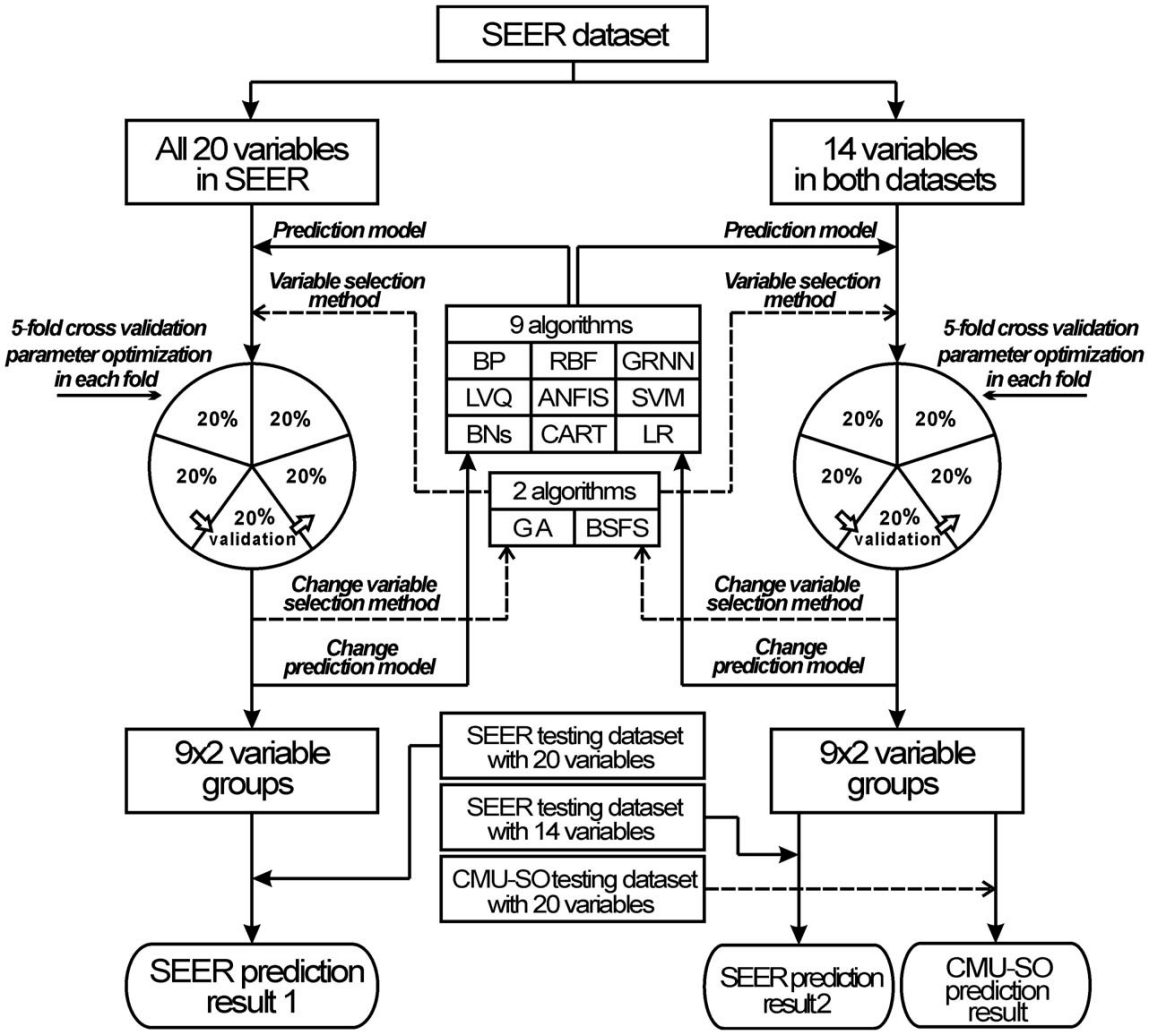
The optimization and prediction system. SEER dataset prediction result A represents the 9*2 predictive results trained by nine data mining algorithms together with two variable selection methods and tested on the SEER testing dataset with all 20 variables. SEER prediction result B represents the 9*2 predictive results tested on the SEER testing dataset with 14 variables supported by both SEER and CMU-SO datasets. CMU-SO prediction result represents the 9*2 predictive results tested on the CMU-SO testing dataset with 14 variables supported by both SEER and CMU-SO datasets.

To build the best model possible for survival prediction of colorectal cancer, we designed an optimization and prediction system by combining several data mining algorithms or statistical methods. As shown in Figure 1, two sub-datasets were used for model training. Dataset A is a 10000*20 matrix involving all 20 variables in the SEER dataset, and Dataset B is a 10000*14 matrix composed of 14 variables supported by both SEER and CMU-SO datasets. We built models with nine different algorithms: BP, RBF, GRNN, ANFIS, SVM, BNs, NB, CART, and LR. To increase the predictive accuracy, variable selection was another important step for building the model. In this study, we made each algorithm select variables that were suitable for the algorithm based on both a genetic algorithm (GA) and a backward stepwise feature selection (BSFS) method. A GA is a search heuristic that mimics the process of natural evolution. It was used to find the optimum subset of variables for each data mining algorithm based on the results of ‘evaluations’ for all ‘chromosomes’ (variable subsets). A BSFS tested each available input variable using each data mining algorithm. Each variable was dropped from the input list, and a determination was made of the resulting loss of predictive accuracy. Only variables that resulted in significant loss of accuracy when dropped were retained. More details about these two methods have been described elsewhere [8], [26], [27]. To reduce possible bias associated with the random sampling of the training, a 5-fold cross-validation was adopted [16]. In 5-fold cross-validation, the original sample was randomly partitioned into five subsamples. Of the five subsamples, a single subsample was retained as the validation data for testing the model, and the remaining four subsamples were used as training data. The cross-validation process was repeated five times, with each subsample used exactly once as the validation data. Several parameter optimization works were implemented in each fold such as searching the most suitable spread value in the GRNN model. More details are shown in Table 2. Subsequently, 9*2 models were created for each sub-dataset. Finally, we tested models trained by Dataset A on the SEER testing dataset with 20 variables, as well as test models trained by Dataset B on two testing datasets: the SEER testing dataset with 14 variables and the CMU-SO testing dataset with 14 variables. The accuracies of the prediction models were measured using the area under the receiver operating characteristic (ROC) curves (AUC) [6]. We used the method of Hanley & McNeil to compare the difference between two ROC curves [28].

After the predictive scores were calculated, to estimate the clinical practical value of prediction models, we divided the patients into eight sub-groups according to the predictive score and compared the predictive survival rate to the real-world survival rates.

### Software and Programs

The system was implemented in Matlab R2009a (MathWorks, Natick, MA). The SVC function in the LIBSVM program (version 2.89) was used to build the SVM model [29]. The BNs model was built by Bayes Net Toolbox (version 1.0.7) [30]. The variable selection based on GA was implemented by the genetic algorithm optimization toolbox (GAOT) [26].

## Results

Based on the GA and BSFS methods, the results of the variable selection on the SEER dataset with 14 and 20 variables are displayed in Tables S1 and S2 separately. *Age at diagnosis*, *EOD 10-extent* and *Regional nodes positive* were selected most often.

**Figure 2:**
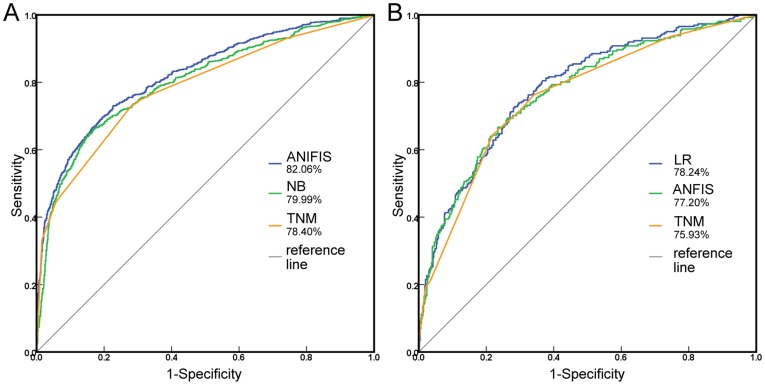
The ROC curve from two different testing datasets. **A.** Comparison of the predictive accuracy of three prognostic models: ANFIS together with GA, NB together with BSFS and the AJCC 7^th^ TNM staging system using SEER testing dataset with 14 variables as a testing dataset. **B.** Comparison of the predictive accuracy of three prognostic models: LR together with BSFS, ANFIS together with GA and the AJCC 7^th^ TNM staging system using the CMU-SO testing dataset as a testing dataset.

The AUCs of nine algorithms were calculated by testing prediction models on the SEER dataset with 14 or 20 variables (Table 3). In the test with 14 variables, based on the best variable selection method, although all algorithms performed better than the AJCC TNM staging system (TNM, AUC = 78.40%; p<0.05), the difference was not great considering the overlapping 95% confidence interval. There were no significant difference in the predictive accuracies of six algorithms (BP, SVM, ANFIS, RBF, GRNN, LR; p>0.05). The ROC curves of ANFIS together with GA and NB together with BSFS which two had the highest and the lowest AUC among nine algorithms are displayed in Figure 2A. The selected variables for ANFIS together with GA were *Age at diagnosis*, *Race/ethnicity*, *Sex*, *Grade*, *EOD 10 – size*, *EOD 10 – extent*, *Regional nodes examined*, *Regional nodes positive*, *Surgery of primary site*, and *Surgery of other reg/dis sites*. The predictive accuracy of the models trained by the SEER dataset with 20 variables was similar with to that trained by the SEER dataset with 14 variables (p>0.05).

**Table 3:** AUC^a^ calculated by testing prediction models on SEER^b^.

	with 20 variables				with 14 variables				*P* &
	AUC	95% confidence interval	variable selection^c^	*P* **	AUC	95% confidence interval	variable selection	*P* **	
BP	81.72%*	79.99%–83.84%	BSFS^d^	<0.001	82.06%*	80.14%–83.99%	GA	<0.001	0.866
CART	80.08%	78.05%–82.10%	BSFS	0.007	80.11%	78.09%–82.13%	BSFS	0.005	0.866
SVM	81.34%	79.37%–83.32%	BSFS	<0.001	81.46%	79.50%–83.42%	Global	<0.001	0.998
ANFIS	81.97%	80.03%–83.89%	GA^e^	<0.001	82.06%	80.15%–83.97%	GA	<0.001	0.811
RBF	81.67%	79.73%–83.63%	GA	<0.001	82.06%	79.92%–83.78%	Global	<0.001	0.620
GRNN	80.98%	78.99%–82.96%	GA	<0.001	80.83%	78.84%–82.81%	BSFS	<0.001	0.625
LR	81.44%	79.47%–83.38%	Global^f^	<0.001	81.42%	79.46%–83.37%	Global	<0.001	0.877
NB	80.10%	78.07%–82.09%	GA	0.003	79.99%	77.96%–82.03%	BSFS	<0.001	0.766
BNs	80.18%	78.16%–82.20%	GA	<0.001	80.25%	78.23%–82.26%	GA	<0.001	0.428
TNM	78.40%	76.28%–80.51%			78.40%	76.28%–80.51%			

AUC^a^: area under the receiver operating characteristic curves.

SEER^b^: National Cancer Institute's Surveillance, Epidemiology, and End Results.

variable selection^c^: the variable selection method which has the highest AUC.

BSFS^d^: variable selection using backward stepwise feature selection.

GA^e^: variable selection using genetic algorithms.

Global^f^: without variable selection.

* : median AUC of 15 tests.

** : comparing the AUC of prediction models with TNM staging system.

& : comparing the AUC of prediction models with 20 variables to that with 14 variables.

The AUCs of nine algorithms were also calculated by testing prediction models on the CMU-SO dataset (Table 4). Although all algorithms obtained larger AUC than the AJCC TNM staging system, the difference of AUC between AJCC TNM staging system and five algorithms, including BP, CART, SVM, ANFIS, and RBF, was not statistically significant (p>0.05). Furthermore, the difference of AUC between AJCC TNM staging system and all nine algorithms is not great considered the overlapping 95% confidence intervals. The ROC curves of LR together with BSFS and ANFIS together with GA which two had the highest and the lowest AUC among nine algorithms are displayed in Figure 2B. The selected variables for LR together with BSFS were *Age at diagnosis*, *Race/ethnicity*, *Sex*, *Grade*, *EOD 10 – extent*, *Regional nodes examined*, and *Regional nodes positive*. There were no significant differences in the predictive accuracies for all algorithms (p>0.05).

**Table 4:** AUC^a^ calculated by testing prediction models on CMU-SO^b^.

	AUC	95% confidence interval	variable selection^c^	*P* **
BP	78.15%*	75.01%–81.10%	GA^d^	0.074
CART	77.29%	73.73%–80.84%	BSFS^e^	0.209
SVM	77.95%	74.47%–81.44%	Global^f^	0.115
ANFIS	77.20%	73.64%–80.76%	GA	0.410
RBF	77.22%	73.67%–80.76%	GA	0.386
GRNN	78.24%	74.77%–81.70%	GA	0.004
LR	78.24%	74.82%–81.67%	BSFS	0.044
NB	78.19%	74.69%–81.69%	BSFS	0.005
BNs	77.90%	74.41%–81.39%	Global	0.013
TNM	75.93%	72.29%–79.57%		

AUC^a^: area under the receiver operating characteristic curves.

CMU-SO^b^: A dataset collects clinical information from Department of Surgical Oncology at the First Hospital of China Medical University.

variable selection^c^: the variable selection method which has the highest AUC.

Global^d^: without variable selection.

GA^e^: variable selection using genetic algorithms.

BSFS^f^: variable selection using backward stepwise feature selection.

* : median AUC of 15 tests.

** : comparing the AUC of prediction models with TNM staging system.

**Figure 3:**
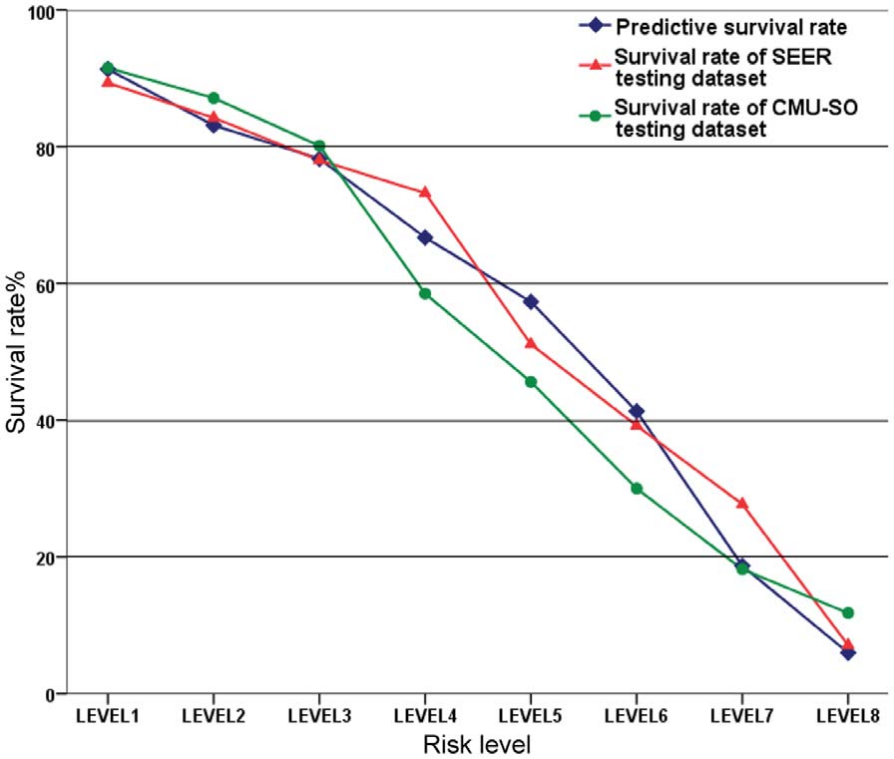
Survival rates at eight risk levels. Comparative result between predictive survival rates to the real-world survival rates at eight different risk levels. The predictive survival rate is based on a predictive model built by LR together with BSFS.

The patients of the CMU-SO dataset were divided into eight groups based on the predictive scores calculated by the LR together with BSFS, which was the combination method with the highest AUC in our study (Figure 3).

## Discussion

Over the past decades, many studies have used data mining technology to predict the 5-year colorectal cancer survival rate [7]–[11], and also to point out superior performance when compared with TNM staging [6]. However, to our knowledge, there are no answers to several questions: Is data mining technology superior in prediction compared to the latest 7^th^ edition of TNM staging? With numerous data mining algorithms, which algorithm is more suitable for prediction of 5-year colorectal cancer survival? When training data and forecast data are from different sources, can the data mining model still be accurate?

To solve these problems, we need an optimization and prediction system that includes common data mining algorithms and we need to evaluate the accuracy of each algorithm. Data mining technologies have two components. One is the algorithm, and we adopted nine algorithms, including nearly all common algorithms. Another is the variables used to construct the model, and a variable selection may be needed by some algorithms. In this study, for each prediction algorithm, we used both the GA and BSFS methods to select suitable variables. In order to reduce possible bias associated with the random sampling of the training, a 5-fold cross-validation was adopted. And then, a parameter optimization program was implemented to increase the accuracy of each prediction model in each fold. (Figure 1)

When prediction models were tested on the SEER dataset, after variable selection and parameter optimization, all algorithms had advantages over the TNM staging system, but the differences were not great (Table 3). This was different from the study of Burke [6], in which they proposed the ANNs were significantly more accurate than the TNM staging system. One possible reason for this was the predictive accuracy of the latest 7^th^ edition of TNM staging was increased compared with the previous edition involved in the study of Burke. Another important finding is that, there were no significant difference in the predictive accuracies of six algorithms (BP, SVM, ANFIS, RBF, GRNN, LR; p>0.05) and the predictive accuracies of the other three algorithms (CART, NB, BNs) were lower. This was different from the study of Grumett [9], in which the accuracy of LR was significantly lower than BP, but was similar to the study of Anderson [11], in which the LR and BP methods outperformed CART and the difference between the accuracy of BP with LR was minimal.

When prediction models were tested on the CMU-SO dataset, there were no significant differences in the predictive accuracies of all the algorithms (p>0.05). The difference of AUC between AJCC TNM staging system and the other five algorithms, including BP, CART, SVM, ANFIS, and RBF, was not statistically significant. Although four of the data algorithms (GRNN, LR, NB, BNs) had an advantage over the TNM staging system, the accuracy was also decreased compared with the test on the SEER dataset. Also, the difference was minor considering the 95% confidence interval (Tables 3, and 4). One possible reason for the lower accuracy was that the race of patients in the CMU-SO dataset was different from that in the SEER dataset. Another possible reason was that different institutions have their own internal audit systems and there was minor difference in the judgment standards for some pathological factors. The differences in the institutions also had negative effects on the predictive accuracy of the TNM staging system. However, the TNM staging system has a simpler model and a relative uniform, worldwide accepted criterion, which made the effects much smaller. A prediction model built by a public dataset is usually needed, such as the SEER dataset used in our study, because it is hard for a single institution to obtain a dataset with large samples. Therefore, the analysis in which the training and testing data were from different sources, such as the test on the CMU-SO dataset in this study, may be more realistic.

Although algorithms of data mining were verified to be slightly more accurate than the TNM staging system for survival prediction of colorectal cancer, we do not wish to claim that the TNM staging system will be replaced by data mining, because the TNM staging system was almost as accurate in predicting the 5-year colorectal cancer survival as the best data mining methods. Furthermore, compared to data mining, the TNM staging system is easier to use and its reproducibility is obviously good. For patients in different stages, standardized treatment of colorectal cancer according to the guidelines will be quickly chosen. However, in the future, a personalized treatment may ask for a more accurate staging system. Therefore, a more complicated staging system based on a data mining method might be needed.

To increase the predictive accuracy of data mining, several studies put the focus on the selection of algorithms [9], [11]–[13]. In this study, we found that there were no significant differences in the predictive accuracies of most algorithms when prediction models were tested on the SEER dataset and there were no significant difference in the predictive accuracies of all algorithms when prediction models were tested on the CMU-SO dataset. Maybe an algorithm that was not included will have better performance, but we venture to think that the improvement will be quite small, considering that we have used nearly all of the common algorithms.

Beyond algorithms, another important component for the data mining technique is the variables included in the models. In this study, the latest version of TNM staging system is sufficiently good that the remaining variables, not related to stage, do not contribute much, significant to what TNM staging system can now do. However, the variables included by SEER dataset were not sufficient. It has been reported that some molecular variables, such as KRAS, have been shown to serve as very powerful predictive indicators, and there has been a trend towards using a dataset that includes both clinical and molecular variables [5]. Therefore, rather than searching for another algorithm, building a large dataset with more variables, especially some molecular variables, may be more effective at present. In addition, it is also important to make a criterion for recording additional variables to decrease the differences in the judgment standard of pathological factors.

Furthermore, as Delen proposed [16], the prediction will be based on a system built by data mining algorithms available to the general public via a website and several algorithms that have been shown to fit the colorectal cancer dataset in this study can be adopted, such as LR. We made a preliminary exploration based on the LR together with BSFS, which was the combination method with the highest AUC in our study (Figure 3).

We conclude that, the TNM staging system is simple and practical at present, and data mining methods are not accurate enough to replace the TNM staging system in colorectal cancer survival prediction. Furthermore, there were no significant differences in the predictive accuracies of all algorithms when the data was from different sources. Building a large dataset including more variables may be important for the elevation of predictive accuracy.

## Supporting Information

Table S1 **Variable selection result on SEER dataset with 14 variables using genetic algorithm and backward stepwise feature selection.** The result of variable selection based on SEER dataset with 14 variables is presented. Both genetic algorithm and backward stepwise feature selection are used.
(DOC)

Table S2 **Variable selection result on SEER dataset with 20 variables using genetic algorithm and backward stepwise feature selection.** The result of variable selection based on SEER dataset with 20 variables is presented. Both genetic algorithm and backward stepwise feature selection are used.
(DOC)
