# Supplementary material for: Which Is a More Accurate Predictor in Colorectal Survival Analysis? Nine Data Mining Algorithms vs. the TNM Staging System
Source: PLoS One. 2012 Jul 25;7(7):e42015. doi: 10.1371/journal.pone.0042015 (PMC3404978; doi:10.1371/journal.pone.0042015)
Supplement: Table S1 — Variable selection result on SEER dataset with 14 variables using genetic algorithm and backward stepwise feature selection. The result of variable selection based on SEER dataset with 14 variables is presented. Both genetic algorithm and backward stepwise feature selection are used. (DOC) [file pone.0042015.s001.doc]

**Table S1 Variable selection result on SEER dataset with 14 variables using genetic algorithm and backward stepwise feature selection**

|  | BP | CART | SVM | ANFIS | RBF | GRNN | LR | NB | BNs |
| --- | --- | --- | --- | --- | --- | --- | --- | --- | --- |
| Age at diagnosis | G**^c^** B**^d^** | G B | G B | G B | G B | G B | G B | G B | G B |
| Race/ethnicity | G B |  | G B | G B | B | B | G B |  | B |
| Sex |  | B | G B | G B | B |  | G B | G B | B |
| Primary Site | B | B |  |  |  |  | G | B |  |
| AJCC^a^ stage 7^th^ | G B | G B |  |  |  | G | G | B | G B |
| Grade |  | B | G | G | G B |  | G B |  | B |
| EOD^b^ 10 – size | B |  |  | G | G B | G |  |  | G |
| EOD 10 – extent | G B | G B | G B | G B | G B | G B | G B | G B | G B |
| Regional nodes examined | G B | G | G B | G B | G B | G | G B |  | B |
| Regional nodes positive | G B | G B | G B | G B | G B | G B | G B | G B | B |
| Number of primaries | G B |  |  |  | G | B |  |  | G |
| Surgery of primary site | G | G | G | G |  | B | G |  | G |
| Surgery of oth reg/dis sites |  |  |  | G |  |  |  |  |  |

**AJCC^a^: American Joint Committee on Cancer**

**EOD^b^: SEER extent of disease**

**G^c^: the variable was selected by the genetic algorithm**

**B^d^: the variable was selected by the backward stepwise feature selection**
