# Supplementary material for: Which Is a More Accurate Predictor in Colorectal Survival Analysis? Nine Data Mining Algorithms vs. the TNM Staging System
Source: PLoS One. 2012 Jul 25;7(7):e42015. doi: 10.1371/journal.pone.0042015 (PMC3404978; doi:10.1371/journal.pone.0042015)
Supplement: Table S2 — Variable selection result on SEER dataset with 20 variables using genetic algorithm and backward stepwise feature selection. The result of variable selection based on SEER dataset with 20 variables is presented. Both genetic algorithm and backward stepwise feature selection are used. (DOC) [file pone.0042015.s002.doc]

**Table S2 Variable selection result on SEER dataset with 20 variables using genetic algorithm and backward stepwise feature selection**

|  | BP | CART | SVM | ANFIS | RBF | GRNN | LR | NB | BNs |
| --- | --- | --- | --- | --- | --- | --- | --- | --- | --- |
| Age at diagnosis | G**^d^** B**^e^** | G B | G B | G B | G B | G B | G B | G B | G B |
| Race/ethnicity |  | B | G B | G B | G B |  | G B | B | G |
| Sex | B |  | G B |  |  |  | G B | G B |  |
| Primary Site | B | B |  |  |  |  |  | G B | G |
| AJCC^a^ stage 7^th^ | B | G B |  |  |  | G | G B | B | G B |
| Grade | G |  | G |  | G |  | G B | B | G |
| EOD^b^ 10 - size |  |  |  |  |  |  |  |  |  |
| EOD 10 - extent | G B | B | G B | G | G | G | G B | G B |  |
| EOD 10 - nodes | G |  |  | G | G |  |  |  |  |
| Regional nodes examined | G B | G | G B | G | G B | G | G B | G |  |
| Regional nodes positive | G B | G B | G B | G B | G B | G B | G B | G B | G B |
| SEER historic stage A |  |  |  |  | B |  |  |  | B |
| SEER summary stage 1977 |  | G |  | B | B | B |  | G | G |
| Histologic Type ICD-O-3^c^ |  |  |  |  | G |  |  |  |  |
| Number of primaries |  |  |  | G |  |  |  |  |  |
| First malignant primary indicator |  | G |  | G |  |  | G B | G | B |
| Radiation sequence with surgery |  |  |  | G | G |  | G |  |  |
| Surgery of primary site |  | G | G | G |  |  |  |  |  |
| Surgery of oth reg/dis sites |  |  |  |  | G |  |  |  | G |

**AJCC^a^: American Joint Committee on Cancer**

**EOD^b^: SEER extent of disease**

**ICD-O-3^c^: International Classification of Diseases for Oncology Third Revision**

**G^d^: the variable was selected by the genetic algorithm**

**B^e^: the variable was selected by the backward stepwise feature selection**
